# Analysis of the information ability of nurses in county hospitals in northern Henan province: a survey

**DOI:** 10.1186/s12912-023-01416-4

**Published:** 2023-07-28

**Authors:** Xizheng Li, Guirui Ma, Kuihong Qiao, Yuwen Yan, Huizhong Zhang, Yan Zhang

**Affiliations:** 1 Department of Nursing, School of Nursing and Health, Zhengzhou University, No. 101, Science Avenue, Zhengzhou City, 450001 Henan Province China; 2 Department of Nursing, The People’s Hospital of Qi xian, Hebi, China; 3 Department of Nursing, The People’s Hospital of Xun xian, Hebi, China

**Keywords:** County hospital, Information capability, Nursing

## Abstract

**Objective:**

To investigate the status quo of nursing information ability of nurses in county-level hospitals and analyze its influencing factors.

**Methods:**

In June 2022, a total of 303 on-the-job clinical nurses from 3 county-level hospitals in Hebi City, Henan Province were selected as subjects by convenience sampling method. General data questionnaire and self-rating nursing information ability scale were used to investigate them.

**Results:**

The total score of nursing information ability of 303 nurses in county hospitals of Hebi City, Henan Province was (77.72 ± 18.76). There were statistically significant differences in the scores of nursing information ability among different ages, working years, positions, education, marriage, monthly income, whether they had learned computer-related knowledge and skills, and whether they had participated in the learning or training of nursing information system (all p < 0.05).Multiple linear regression analysis showed that age, years, position, monthly income and whether they had learned computer-related knowledge and skills were the main influencing factors of nursing information ability of county-level nurses (all p < 0.05).

**Conclusions:**

The nursing information ability of nurses in county-level hospitals in northern Henan is at a medium level. The government or society should provide training and guidance on nursing information ability, so as to provide more opportunities for nurses in county-level hospitals to participate in and learn nursing information technology to improve their ability.

## Introduction

Nursing information ability refers to the comprehensive ability of knowledge, technology and attitude of nurses in the face of various nursing information activities [1]. Studies have shown that [2], Improving the information ability of nurses can promote providing better quality and safer services for patients, and can also promote the self-development of nurses and improve their sense of professional benefits. before one,With the rapid development of global medical informatization, nurses need to integrate the knowledge of computer science, information science and cognitive science into their work [3], Nursing information ability has become one of the core professional abilities and essential qualities of clinical nurses [4]. According to the Work Plan for Improving the Comprehensive Capacity of County Hospitals (2021–2025) issued by the General Office of the National Health Commission in 2021 [5]. By 2025, at least 1,000 county hospitals in the country will reach the medical service capacity level of tertiary hospitals, emphasizing accelerating the construction of digital health infrastructure in county-level hospitals and improving the conditions of information equipment. Therefore, the new requirements are put forward for the information capacity of nurses in county-level hospitals. However, the relevant research objects of domestic nursing information ability are mainly nursing students and clinical nurses in tertiary hospitals [6], There are few studies on the nursing information ability of nurses in county hospitals.

The innovation of this study was to focus on the information nursing ability of county-level hospital nurses, a long neglected group. This study investigated the nursing information ability of nurses in county-level hospitals to supplement the shortcomings of research on nursing information ability of nurses in grassroots hospitals. This study was aimed to understand the current situation and analyze the influencing factors, in order to provide valuable reference for the development of targeted nursing information ability improvement program of clinical nurses in county hospitals.

## Objects and methods

### Study subjects

**Fig. 1:**
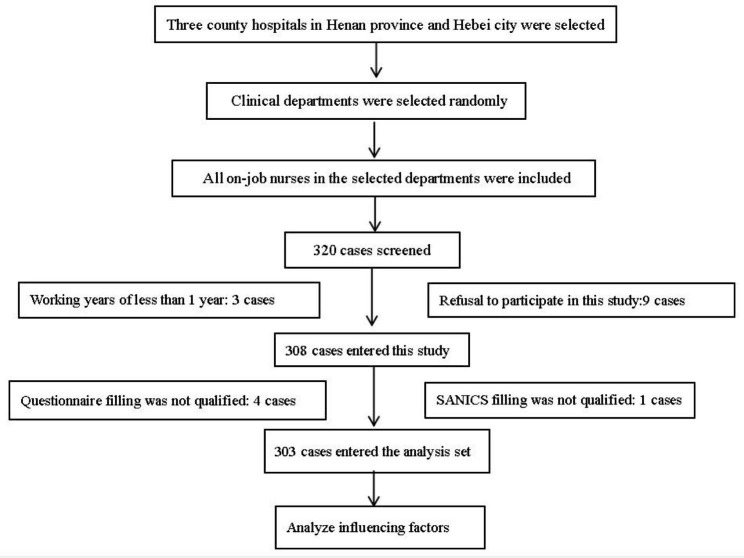
The screening process of the survey objects

From June to September 2022, the stratified sampling method was adopted to randomly select three county hospitals in Henan province and Hebei city, and randomly selected clinical departments in each department, and then all on-job nurses in the selected departments were included as the survey objects. Inclusion criteria were as follows: (1) on-job nurses with nurse practice certificates and working years of more than 1 year; (2) gave informed consent to voluntarily participate in the study. Sample size calculation: According to 5–10 times of the self-assessment table of nursing information capacity (28 items), and considering 20% inefficiency, the maximum sample content of this study = 2810 (1 + 20%) = 336, the minimum content = 285 (1 + 20%) = 168, the sample content is 303 cases to meet the sample size requirements. All of the survey respondents gave their informed consent. 320 nurses were screened, excluding nurses who did not meet the standards, and 308 nurses entered this study. In the end, 303 qualified nurses completed the data collection and were included in the analysis set for analysis of influencing factors. The screening process is shown in Fig. 1.

### Study methods

#### Research tools

1. General data questionnaire: 11 questions, including gender, age, working years, employment method, professional title, position, position, educational background, marital status, monthly income, whether they have learned computer-related knowledge and skills, and whether they have participated in the study or training of nursing information system.
2. Self-evaluation scale of nursing information ability.

The self-assessment of nursing informatics competencies scale(SANICS) was developed by YOON et al. in 2009 [7]. This scale is often used to evaluate nurses’ information technology skills and has good reliability and validity [10]. Several countries such as South Korea, Canada, and China have adapted this scale across cultures [8–10]. Yu Zijuan Yu was used in this study [10]. The Chinese version of nursing information ability self-assessment form. Including clinical information role (5 items), basic computer knowledge and skills (11 items), application ability of computer skills (4 items), clinical information attitude (4 items) and wireless device skills (4 items), 5 dimensions, a total of 28 items. Using Likert5 scoring method, the value was 1 to 5 points from “not competent” to “reaching expert level”. The total score ranged from 28 to 140, the middle level was 84, the higher the score, a higher level of nursing information ability. The SANICS α coefficient is 0.931, the retest reliability is 0.883, and the content validity is 0.950 [11]. The Cronbach’s α coefficient in this study was 0.910.

#### Data collection method

All participant were required to fill out a general data questionnaire and SANICS, both in paper form. Before filling out, participants need to read the filling rules and fill out the questionnaire and SANICS according to the filling rules. After the participants fill out, a designated research team member will inspect it to ensure that there were no missing options before retrieving the questionnaire and SANICS. Any omissions or logical errors are considered unqualified. The purpose, significance and confidentiality of the study were explained to the subjects before the study, and the questionnaire was issued after obtaining the informed consent of the subjects.

#### Observation indicator

The Observation indicator was the effect factors of nurses’ nursing information ability. Possible influencing factors include gender, age, working years, employment method, professional title, position, position, educational background, marital status, monthly income, whether they have learned computer-related knowledge and skills, and whether they have participated in the study or training of nursing.

### Statistical methods

The results were entered and analyzed using SPSS 25.0, and two results were recorded to ensure the accuracy of the data. The general data of the research subjects were statistically described by means, standard deviation, frequency, and composition ratio, t-test, one-way variance analysis of county hospitals, and the main influencing factors were analyzed by multiple linear regression, with the test level α = 0.05.

## Results

### Nurses’ information technology skills

The SANICS score showed that the total score of 303 nursing staff in this survey was (77.72 ± 18.76). See Table 1. Item scores are shown in Tables 2 and 3.

**Table 1:** Score of nursing information ability of 303 caregivers (score, x ± s)‾

project	score	Project average score
Total table score	77.72 ± 18.76	2.78 ± 0.67
Clinical Information Role	11.94 ± 3.35	2.39 ± 0.67
Basic computer knowledge and skills	26.64 ± 6.50	2.42 ± 0.59
Application ability of computer skills	7.63 ± 2.59	1.91 ± 0.65
Wireless device skills	8.90 ± 2.64	2.23 ± 0.66
Clinical information attitudes	17.33 ± 3.03	4.33 ± 0.76

**Table 2:** Minimum score of nursing staff entry (score, x ± s)‾

clauses and subclauses	score	sort
Use the application for diagnostic coding	1.81 ± 0.74	28
Develop the test materials using the application program	1.81 ± 0.75	27
Basic troubleshooting in the application	1.95 ± 0.69	26
Identify the basic components of a computer system (e.g., PC, workstation function)	2.07 ± 0.72	25
Data were extracted from the clinical data set	2.21 ± 0.75	24

**Table 3:** Top score of nurse staff entry (score, x ± s)‾

clauses and subclauses	score	sort
Recognize the importance of clinician involvement for the design, selection, implementation, and evaluation of the healthcare system	4.41 ± 0.84	1
Recognize that “Internet plus health” will become more common	4.34 ± 0.85	2
Realizing that computers are only a tool to promote nursing work does not completely replace the human function	4.33 ± 0.82	3
Recognize that it is not only computer programmers who can effectively apply computer technology to nursing work	4.25 ± 0.89	4
Apply electronic communication devices (such as Wifi, mobile broadband or other devices) to exchange information (through data upload, download, etc.)	2.71 ± 0.74	5

### Analysis of the influencing factors of nurses’ nursing information ability

The results of this study show that the differentAge, working years, position, education background, marital status, monthly income, whether orLearning computer-related knowledge and skills, whether to participate in learning or training on nursing information systems are the factors affecting the nursing information ability of nurses in county hospitals, and the differences were statistically significant (all p < 0.05), shown in Table 4.

**Table 4:** Results of univariate analysis of nurse general data and nursing information ability (points, x ± s)‾

project	Number of cases, [n (%)]	Score (score, x ± s)	*t perhaps F*	*p*
sex			−0.536	0.592
man	32(10.6)	79.406 ± 19.22		
woman	271(89.4)	77.52 ± 18.73		
age			3.221	0.029
<25	78(25.7)	73.15 ± 12.57		
25-	90(29.7)	78.46 ± 15.96		
35-	120(39.6)	79.92 ± 22.74		
45-	15(5)	79.60 ± 23.30		
working life			9.180	<0.001
1-	92(30.4)	71.40 ± 12.26		
5-	62(20.4)	77.34 ± 16.52		
10-	120(39.6)	84.30 ± 22.01		
15-	44(9.6)	75.77 ± 19.98		
Employment method			−1.447	0.149
Officially in the	120(39.6)	75.80 ± 18.06		
agreement	183(60.4)	78.98 ± 19.15		
professional ranks and titles			2.654	0.086
nurse	123(40.6)	74.25 ± 14.93		
primary nurse	116(38.2)	81.22 ± 21.78		
nurse-in-charge	60(19.8)	78.07 ± 19.01		
Deputy chief nurse teacher and above	4(1.4)	77.75 ± 10.72		
post			3.327	0.02
general duty nurse	266(87.8)	76.45 ± 18.34		
Ward head nurse	17(6)	86.82 ± 19.06		
supervisor of nursing care	18(6)	85.72 ± 21.50		
Associate Director	2(0.2)	93.00 ± 7.07		
record of formal schooling			8.423	<0.001
special school	9(3)	69.56 ± 27.40		
junior college	106(35)	72.46 ± 16.79		
undergraduate course	188(62)	81.08 ± 18.76		
Master’s degree or above	0(0)			
marital status			5.095	0.017
married	181(59.7)	80.11 ± 20.63		
unmarried	114(37.6)	73.64 ± 14.35		
other	8(2.7)	81.86 ± 21.80		
monthly income			17.944	<0.001
< 3000	76(25.1)	72.74 ± 15.56		
3000-	146(48.2)	73.47 ± 16.04		
5500-	78(25.7)	89.21 ± 20.38		
>8000	3(1)	112.67 ± 13.65		
Learn computer-related knowledge and skills			6.589	<0.001
yes	190(62.7)	82.52 ± 19.48		
deny	113(37.3)	69.66 ± 14.29		
Participate in learning or training on aspects of nursing information systems			3.951	<0.001
yes	221(72.9)	80.06 ± 19.21		
deny	82(27.1)	71.42 ± 15.95		

### Multiple linear regression analysis of nurses’ nursing information ability

The score of the self-rating scale of nursing information capacity was used as the dependent variable to age, length of years, position, highest degree, average monthly income, whetherLearning computer-related knowledge and skills, participating in learning or training on nursing information systems as independent variables, using a stepwise multiple linear regression analysis (α in = 0.05, α out = 0.10). The results showed that age, years, position, monthly income, whether they had learned computer-related knowledge and skills were the influencing factors of nursing information ability (p < 0.05), as shown in Table 5.

**Table 5:** Multiple linear regression analysis of the factors influencing the nurses’ information ability

variable	β	S.E.	β’	t	P
constant term	63.039	5.184	-	12.159	< 0.001
Age of 35-	-6.569	2.486	-0.172	-2.642	0.009
Years of service: 10-	20.118	3.313	0.511	6.073	< 0.001
Head nurse of the duty ward	8.422	3.777	0.103	2.230	0.027
Monthly income of 5,500-	12.821	2.194	0.299	5.844	< 0.001
8000-	43.487	8.872	0.230	4.902	< 0.001
Learn computer-related knowledge and skills	12.385	1.775	0.320	6.976	< 0.001

## Discussion

### The information capacity of nurses in county-level hospitals is at a lower medium level

The results of this survey showed that 303 county hospital nurses had nursing information ability scores (77.72 ± 18.76), which was at the lower medium level. The reasons may be as follows: the subjects of this survey have bachelor’s degree or below, mainly junior college degree. At present, higher vocational colleges lack teachers with nursing informatics professional background, and informatics related course resources are limited, lack of computer operation practice opportunities, practical tasks such as literature retrieval and paper writing [12]. Therefore, the information ability of nurses in county hospitals mainly with junior college degree is weak. In addition, the majority of people involved in the survey about nursing information system learning or training (72.9%), but the nursing information ability score is not high, the side reflects the current hospital nurses nursing information ability training may be fragmented and content not practical or in-depth, cannot meet the needs of nurses actual training [13]. In addition, the scores of each dimensions show that the dimension of nursing information attitude is the highest. The reason may be that most respondents have realized the important role of “Internet + nursing” and put forward new requirements for the computer skills of medical staff in the era of big data [14]. At the same time, the computer application ability dimension score of the respondents of this survey is low, which indicates that the computer practice operation ability of the survey subjects is weak. The reason may be the lack of systematic and standardized nursing information for nurses in county-level hospitals, and there are problems such as emphasizing theory over practice, and single training form [15]. Therefore, county-level hospitals should formulate corresponding training content for the weak links of nurses’ computer application ability to increase their practical operation opportunities [16]. It was helpful to consider to develop primary, intermediate and advanced information ability step training program to quickly and effectively improve the information ability level of clinical nurses.

### Analysis of influencing factors of nursing information ability of nurses in county hospitals

According to the results of this survey, the factors influencing the nursing information ability of nurses in a county hospital in northern Henan province include age, working years, position, monthly income, and whether they have learned computer-related knowledge and skills. The specific analysis is provided as follows:

#### Age

This study showed that the clinical working nurses aged between 35 and 44 years had higher comprehensive scores of nursing information ability. The reasons may be: nurses at this age are the backbone of the hospital, with more learning resources and opportunities, relatively stable working environment, strong professional ability and relatively small work pressure [17]. Studies have shown that nurses are burdened by the constant need to redefine their nursing expertise to provide care in digital environments [18].Particularly the first years of professional nursing practice are known to be stressful with a lot of learning and adaptation ^[19, 20]^. The older nurses use the Internet to obtain knowledge, resulting in their weak clinical nursing information ability. Therefore, the relevant departments of county hospitals should give full play to the advantages of nurses aged 35–44, and learn the basic knowledge of computer application skills at different levels according to the clinical experience of nurses with low seniority. At the same time, it is suggested that high seniority nurses and young nurses form a point-to-point support relationship, form a study group, learn from each other in time, and jointly improve the information ability level of clinical nurses.

#### Years of service

This study shows that nurses in county hospitals with working years of 10–14 years have strong nursing information ability. The analysis reasons may be that nurses (working years < 10 years) mainly focus on the evaluation of clinical nursing practice, basic nursing, specialized nursing knowledge and skills implementation, Less involved to the questionnaire questions involved"Use the application for diagnostic coding”“Data were extracted from the clinical data set"And other content, its scientific research and exploration consciousness is relatively weak; andSenior nurses (working years > 15 years) are mainly responsible nurses in administrative management or overall nursing, focusing on the completion of medical tasks, and less involve “using application programs to develop test materials”, so their scores are low. And Staggers et al.^[21]^ Nurses were divided into four levels: new nurses, experienced nurses, informatics experts and informatics innovators. It was suggested that the post-graded training mode in the hospital should be formulated according to different working years, so as to accelerate the improvement of nurses’ information ability. In addition, it pays attention to the cultivation of nursing scientific research consciousness and ability and information innovation ability. By carrying out nursing research in big data and data extraction method training, it guides nurses in county hospitals to continuously apply and innovate nursing informatization ways in their work, and gradually grow in the practice of nursing informatization.

#### Position

This study showed that the ward head nurse had higher scores in nursing information ability. Due to the needs of their own management tasks, the head nurse of the ward will use the nursing information equipment more deeply and extensively to promote the improvement of their nursing information ability. Studies have shown that^[22]^, The head nurse in the nursing information system of nursing quality, nursing assessment, nursing scheduling, and daily affairs and so on various aspects of management, need to frequently use nursing information equipment and application to accelerate the development of its nursing information ability, and county hospital ordinary nurses mainly focus in clinical line, bear the whole nursing from admission to discharge. Therefore, hospitals should build a training system with clear levels according to the different nursing information abilities and positions of nurses, formulate training programs that meet the needs of different positions, improve the practical ability of nurses in computer application, and increase the opportunities for ordinary clinical nurses to learn nursing information knowledge. The results of this survey show that most head nurses have realized the importance of nursing information ability, and should play their own role models to drive the improvement of the nursing information ability of the overall nursing team. In addition, nursing information-related scholars can also further explore the evaluation criteria of nursing information ability for the duty nursing staff in county-level hospitals, so as to meet the training requirements of compound nursing talents.

#### Monthly income

This study showed that the average monthly income was an influencing factor of the nursing information competency score. The higher the income respondents the stronger its nursing information ability, the reason may be: high income nurses professional identity is relatively higher than low income^[23]^, the former is more likely to be willing to actively learn literature retrieval, retrieval strategy knowledge, may contact information technology products, prompting them to learn to update information technology, information ability to improve^[24]^. Based on the results of this survey, it is suggested to further mobilize the enthusiasm of nurses in county hospitals and continuously enhance their professional identity. Guide them to pay attention to the process of nursing information construction, and constantly improve the personal nursing information ability. In addition, it is reported that “Internet + nursing service” can increase the income of nurses and enhance the professional identity of nurses. Therefore, county-level hospitals may quickly promote the “Internet + nursing service” business through publicity and expand the improvement of nurses’ nursing information ability.

#### Have learned computer-related knowledge and skills

This study showed that nurses who had received computer-related knowledge and skills learning had higher scores on the self-assessment form of nursing information ability. This is consistent with study of Elsayed WA [25]. Studies have shown that [26], With the network development of the working environment, hospitals have an increasing demand for nursing talents with computer operation ability, and their computer operation level is also higher, eventCounty-level hospitals can consider combining the information development of the hospital with the nursing information ability of nurses, and encourage them to participate in the visit and study of superior hospitals with higher level of information construction [27], To create more training opportunities to improve their information capabilities. In addition, whether the computer operation can be listed as a basic nursing skills, specialized skills of daily skills, compulsory examination items, May play furtherThe role of computer training, so as to promote the nursing information ability of nurses in county hospitals.

## Conclusion

This study found that the nursing information ability of nurses in county hospitals urgently needs to be improved, and the influencing factors include age, working years, position, monthly income, and whether they have learned computer-related knowledge and skills. Provide targeted training based on the age and length of service of nurses. Pay attention to the nursing information abilities of young and elderly nurses, and receive guidance and assistance from nurses aged 35–44.It was also important to increase the income of nurses and strengthen the learning of computer related knowledge and skills. Due to the time and region limitation, this study only investigated the current situation of clinical nurses in some county hospitals in northern Henan province, and the deep influencing factors need to be explored. Therefore, in the follow-up research, multi-center investigation can be considered, and combined with qualitative research methods to deeply understand the deep influencing factors of the nursing information ability of nurses in county hospitals in different regions, so as to provide some reference for the targeted information ability intervention of nurses in county hospitals.

## Data Availability

All data generated or analysed during this study are included in this article. Further enquiries can be directed to the corresponding author.
